# Family Clustering of Avian Influenza A (H5N1)

**DOI:** 10.3201/eid1111.050646

**Published:** 2005-11

**Authors:** Sonja J. Olsen, Kumnuan Ungchusak, Ly Sovann, Timothy M. Uyeki, Scott F. Dowell, Nancy J. Cox, William Aldis, Supamit Chunsuttiwat

**Affiliations:** * International Emerging Infections Program, Nonthaburi, Thailand; † Ministry of Public Health, Nonthaburi, Thailand; ‡ Ministry of Health, Phnom Penh, Cambodia; § Centers for Disease Control and Prevention, Atlanta, Georgia, USA; ¶ World Health Organization, Nonthaburi, Thailand

**Keywords:** avian influenza, H5N1, letter, family clusters

**To the Editor:** The unprecedented epizootic of avian influenza A (H5N1) in Asia poses a serious threat of causing the next global influenza pandemic. H5N1 viruses, to which humans have little or no immunity, have demonstrated the capacity to infect humans and cause severe illness and death (*1**–**4*). Fortunately, these viruses have not yet demonstrated the capacity for efficient and sustained person-to-person transmission, although limited person-to-person transmission was the cause of at least 1 family cluster of cases (*5*). Since family clusters of H5N1 illness may be the first suggestion of a viral or epidemiologic change, we have been monitoring them with great interest.

Through our regional contacts and public sources, we have monitored family clusters and other aspects of H5N1 in Southeast Asia. A cluster was defined as >2 family members with laboratory-confirmed H5N1 or >2 family members with severe pneumonia or respiratory death, at least one of which had confirmed H5N1. To determine if family cluster events had increased over time, we divided the number of cluster events by the total number of days in 2 discrete periods and calculated rate ratios (RR) and 95% confidence intervals (CI). To determine whether the increase in family clustering was attributable to an increase in the number of cases, we divided the number of family units with >2 laboratory-confirmed cases by the total number of family units in the period. Percentage of deaths was also compared.

From January 2004 to July 2005, 109 cases of avian influenza A (H5N1) were officially reported to the World Health Organization (WHO) (*6*). During this time, 15 family clusters were identified (Table). Of the 11 (73%) clusters that occurred in Vietnam, 7 were in northern Vietnam. Cluster size ranged from 2 to 5 persons, and 9 (60%) had >2 persons with laboratory-confirmed H5N1. Cluster 6 in Thailand was well documented and was likely the result of limited person-to-person transmission (*5*). For the other clusters, epidemiologic information was insufficient to determine whether person-to-person transmission occurred. In at least 3 clusters in Vietnam (Table; clusters 5, 7, and 11), >7 days occurred between the onset of the first and the next case, suggesting that simultaneous acquisition from a common source was unlikely. In cluster 11, 2 nurses assisted in the care of the index case-patient and subsequently were hospitalized with severe pneumonia; 1 had laboratory-confirmed H5N1.

**Table:** Family clusters of influenza A (H5N1) in Southeast Asia, January 2004–July 2005*

Cluster	Onset of index case	Country	Age (y)/Sex	Relation to index case	H5N1	Onset	Outcome
1	Dec 03	Vietnam (N)	12/F	Self	+	Dec 25	D
			30/F	Mother	+	Jan 1	D
2	Dec 03	Vietnam (N)	5/M	Self	+	Dec 29†	D
			7/F	Sister	NT	NN	D
3	Jan 04	Vietnam (N)	31/M	Self	NT	Jan 7†	D
			30/F	Sister	+	Jan 10	D
			28/F	Wife	+	Jan 10	R
			23/F	Sister	+	Jan 11	D
4	Jan 04	Thailand	6/M	Self	+	Jan 8	D
			33/F	Mother	NT	Jan 8	D
5	Jul 04	Vietnam (S)	19/M	Self	NT	Jul 23	D
			22/F	Cousin	NT	NN	D
			25/F	Sister	+	Jul 31	D
6	Sep 04	Thailand	11/F	Self	NT	Sep 2	D
			26/F	Mother	+	Sep 11	D
			32/F	Aunt	+	Sep 16	R
7	Dec 04	Vietnam (N)	46/M	Self	+	Dec 26	D
			42/M	Brother	+	Jan 10†	R
			36/M	Brother	+	Not ill	Not ill
8	Jan 05	Vietnam (S)	17/M	Self	+	Jan 10†	D
			22/F	Sister	NN	NN	Unknown‡
9	Jan 05	Vietnam (S)	35/F	Self	+	Jan 14	D
			13/F	Daughter	+	Jan 20	D
10	Jan 05	Cambodia	14/M	Self	NT	NN	D
			25/F	Sister	+	Jan 21	D
11	Feb 05	Vietnam (N)	21/M	Self	+	Feb 14	Unknown‡
			14/F	Sister	+	Feb 23	Unknown‡
			80/M	Grandfather	+	Not ill	Not ill
12	Feb 05	Vietnam (N)	69/M	Self	+	Feb 19	D
			61/F	Wife	+	Not ill	Not ill
13	Mar 05	Vietnam	13/F	Self	NT	Mar 9§	D
			5/M	Brother	+	Mar 12†	R
			Adult/F	Aunt	P	NN	Unknown‡
14	Mar 05	Vietnam (N)	39/M	Self	+	Mar 22†	Unknown‡
			Adult/F	Wife	+	Mar 22†	Unknown‡
			4 mo/NN	Child	+	Mar 22†	Unknown‡
			3/NN	Child	+	Mar 22†	Unknown‡
			10/NN	Child	+	Mar 22†	Unknown‡
15	Jul 05	Indonesia	8/F	Self	+¶	Jun 24	D
			1/F	Sister	NT	Jun 29	D
			38/M	Father	+	Jul 2	D

*D, respiratory death; N, north; NT, not tested; NN, not noted; P, pending; R, recovered; S, south. †Date of hospitalization. ‡Had respiratory symptoms, was hospitalized (unknown for #13), and outcome was unknown. §Date of death. ¶Serologically confirmed; classified as a probable case by the World Health Organization.

Family clusters were slightly more likely to have occurred between December 2004 and July 2005 than in the first year of the outbreak (9 clusters in 243 days or 3.7 per 100 days vs. 6 clusters in 365 days or 1.6 per 100 days, respectively; RR 2.3, 95% CI 0.8–6.3). The difference was similar when the periods were limited to the same 8 months, 1 year apart (RR 1.8, 95% CI 0.6–5.4). Twenty-five (61%) of the 41 patients in the 15 family clusters died; the 7 persons who recovered or were not ill experienced secondary cases.

Family clusters are still occurring; however, they do not appear to be increasing as a proportion of total cases. The proportion of families that were part of a cluster was similar from December 2004 to July 2005 to the proportion in the first year (6/55, 11% vs. 3/41, 7%, respectively, p = 0.7). However, the proportion of deaths dropped significantly, from 32 of 44 (73%) during December 2003 to November 2004, to 23 of 65 (35%) during December 2004 to July 2005 (p<0.0001).

Although reports of H5N1 family clusters slightly increased, the increase was not statistically significant. Nevertheless, we believe any cluster of cases is of great concern and should be promptly and thoroughly investigated because it might be the first indication of viral mutations resulting in more efficient person-to-person spread. Family clustering does not necessarily indicate person-to-person transmission, as it may also result from common household exposures to the same H5N1-infected poultry or from other exposures, such as to uncooked poultry products.

The decrease in proportion of deaths during 2005 is another epidemiologic change that should be monitored closely because it may reflect viral adaptation to the human host. Surveillance for human cases of avian influenza has been intensified in recent months, perhaps resulting in the identification of less severe cases. Alternatively, more widespread laboratory testing may be associated with false-positive results. No evidence to date shows genetic reassortment between H5N1 and human influenza A viruses (*7*). Viruses isolated from case-patients need to be immediately sequenced and characterized in relation to previously circulating viruses to see whether they are evolving.

Recent modeling studies suggest that containing a pandemic at its source may be possible because emergent pandemic viruses may be less transmissible than commonly assumed (*8*), and antiviral treatment and chemoprophylaxis may slow the spread (*9*). Although the logistics of an attempt to contain the beginning of a potential influenza pandemic are formidable, we believe it is not beyond the capability of the modern global public health system. As WHO (*10*) has called for, countries should intensify their pandemic preparedness plans and strengthen international collaborations.

## References

[1] Hien TT, de Jong M, Farrar J. Avian influenza—a challenge to global health care structures. N Engl J Med. 2004;351:2363–5. 10.1056/NEJMp048267 15575048

[2] Centers for Disease Control and Prevention. Cases of influenza A (H5N1)—Thailand, 2004. MMWR Morb Mortal Wkly Rep. 2004;53:100–3. 14961002

[3] Chokephaibulkit K, Uiprasertkul M, Puthavathana P, Chearskul P, Auewarakul P, Dowell SF, A child with avian influenza A (H5N1) infection. Pediatr Infect Dis J. 2005;24:162–6. 10.1097/01.inf.0000151037.25237.1e 15702046

[4] Chotpitayasunondh T, Ungchusak K, Hanshaoworakul W, Chunsuthiwat S, Sawanpanyalert P, Kijphati R, Human disease from influenza A (H5N1), Thailand, 2004. Emerg Infect Dis. 2005;11:201–9. 15752436 10.3201/eid1102.041061 PMC3320461

[5] Ungchusak K, Auewarakul P, Dowell SF, Kitphati R, Auwanit W, Puthavathana P, Probable person-to-person transmission of avian influenza A (H5N1). N Engl J Med. 2005;352:333–40. 10.1056/NEJMoa044021 15668219

[6] World Health Organization. Cumulative number of confirmed human cases of avian influenza A/(H5N1) reported to WHO. Vol. 2005. Geneva: The Organization; 2005. [cited 2005 Sep 22]. Available from http://www.who.int/csr/disease/avian_influenza/country/cases_table_2005_07_27/en/index.html

[7] World Health Organization. Evolution of H5N1 avian influenza viruses in Asia. Emerg Infect Dis. 2005;11:1515–21. 16318689 10.3201/eid1110.050644 PMC3366754

[8] Mills CE, Robins JM, Lipsitch M. Transmissibility of 1918 pandemic influenza. Nature. 2004;432:904–6. 10.1038/nature03063 15602562 PMC7095078

[9] Longini IM Jr, Nizam A, Xu S, Ungchusak K, Hanshaoworakul W, Cummings DA, Containing pandemic influenza at the source. Science. 2005;309:1083–7. Epub 2005 Aug 3. 10.1126/science.1115717 16079251

[10] World Health Organization. Influenza pandemic preparedness and response. Geneva: The Organization; 2005. [cited 2005 Sep 22]. Available from http://www.who.int/gb/ebwha/pdf_files/EB115/B115_44-en.pdf

